# Supplementary material for: Visualization of Aspalathin in Rooibos (Aspalathus linearis) Plant and Herbal Tea Extracts Using Thin-Layer Chromatography
Source: Molecules. 2019 Mar 7;24(5):938. doi: 10.3390/molecules24050938 (PMC6429207; doi:10.3390/molecules24050938)
Supplement: Supplementary file 1 [file molecules-24-00938-s001.pdf]

molecules

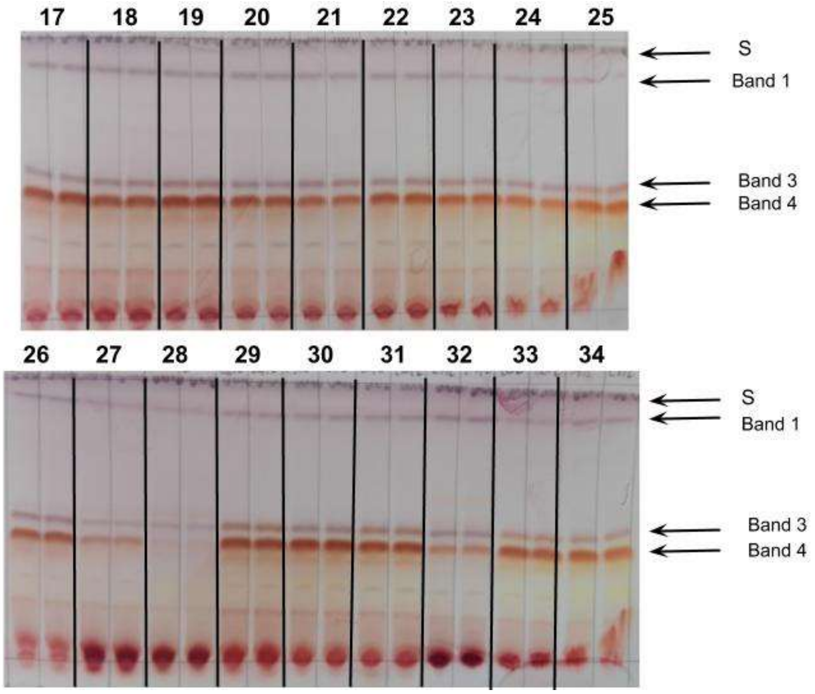

Figure S1: A subset from a total of 109 rooibos ecotypes analyzed with the described TLC method. Plants 17-25,26,29-31,34 = Commercial; plant 27,28,32,33= wild-growing. S: solvent front.

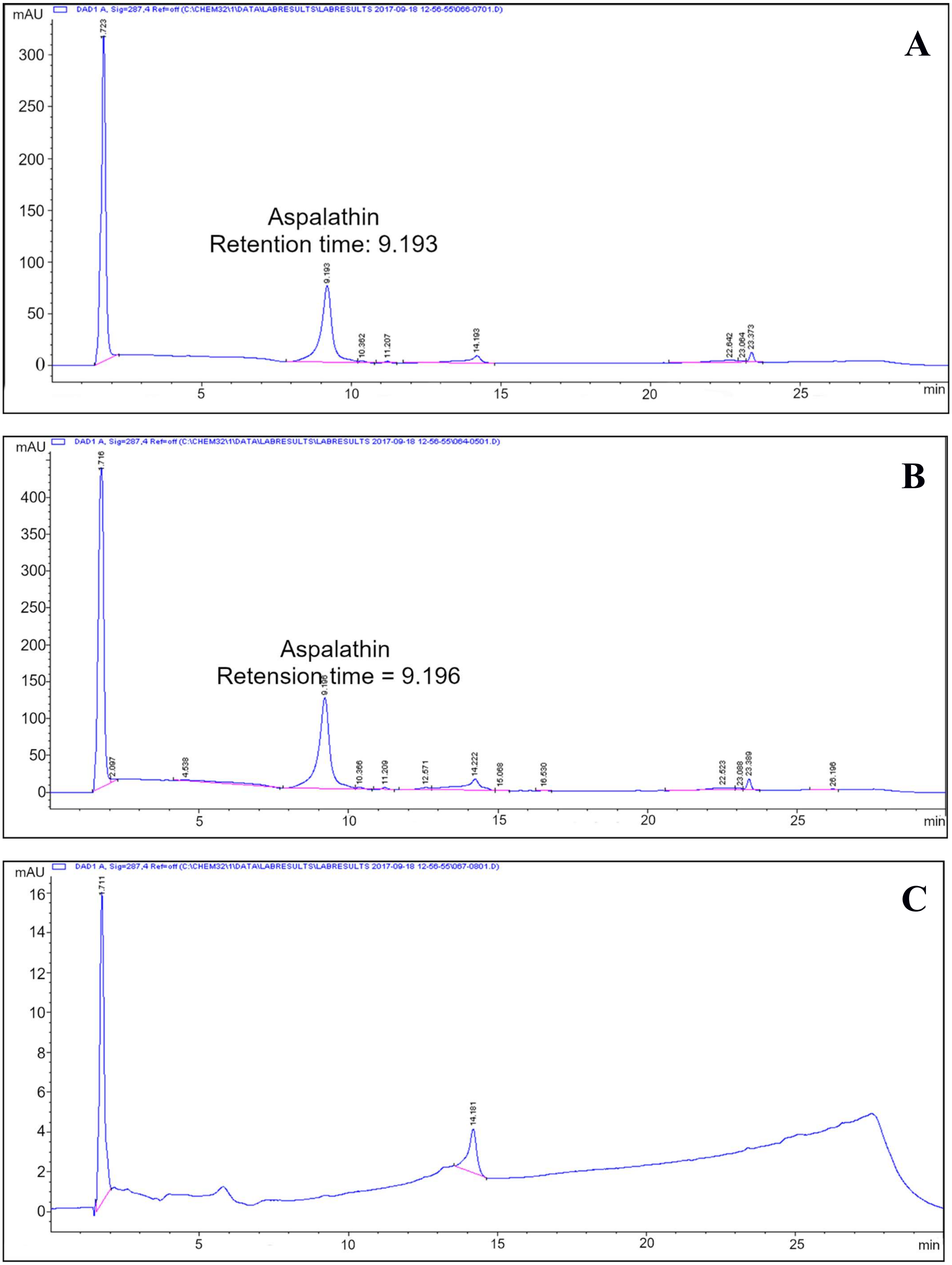

Figure S2: HPLC-DAD and UPLC-MS chromatograms of the preparative TLC extracts at RF = 0.46 suspected to contain aspalathin. The chromatograms A and B are TLC silica extracts from plants

1 and 2, verifying the presence of aspalathin at retention time = 9.1 min. The chromatogram C is a silica extract at RF = 0.46 from a TLC of an aspalathin negative plant 3, verifying no aspalathin at the equivalent position.

Table S1. Description of a subset from the sampled rooibos plants

Plant	Growth types	Location	Region
17	Commercial rooibos plant	S032° 47' 22″ E18° 48' 26″	Clanwilliam
18	Commercial rooibos plant	S032° 47' 22″ E18° 48' 26″	Clanwilliam
19	Commercial rooibos plant	S032° 40' 06″ E18° 38' 10″	Clanwilliam
20	Commercial rooibos plant	S032° 40' 06″ E18° 38' 10″	Clanwilliam
21	Commercial rooibos plant	S032° 40' 07″ E18° 38' 09″	Clanwilliam
22	Commercial rooibos plant	S032° 40' 07″ E18° 38' 09″	Clanwilliam
23	Commercial rooibos plant	S032° 40' 07″ E18° 38' 09″	Clanwilliam
24	Commercial rooibos plant	S031º 43' 09″ E019º 08' 25″	Nieuwoudtville
25	Commercial rooibos plant	S031º 43' 09″ E019º 08' 25″	Nieuwoudtville
26	Commercial rooibos plant	S031º 43' 09″ E019º 08' 25″	Nieuwoudtville
27	Nieuwoudtville sprouter	S031º 42' 13″ E019º 07' 26″	Nieuwoudtville
28	Nieuwoudtville sprouter	S031º 42' 13″ E019º 07' 27″	Nieuwoudtville
29	Commercial rooibos plant	S031º 43' 17″ E019º 07' 29″	Nieuwoudtville
30	Commercial rooibos plant	S031º 43' 18″ E019º 07' 31″	Nieuwoudtville
31	Commercial rooibos plant	S031º 43' 18″ E019º 07' 32″	Nieuwoudtville
32	Nieuwoudtville sprouter	S031º 45' 48″ E019º 07' 54″	Nieuwoudtville
33	Black type	S031º 45' 48″ E019º 07' 54″	Nieuwoudtville
34	Commercial rooibos plant	S031º 47' 22″ E019º 06' 51″	Nieuwoudtville
